# The Benefit of Thrombectomy in Patients With Low ASPECTS Is a Matter of Shades of Gray—What Current Trials May Have Missed

**DOI:** 10.3389/fneur.2021.718046

**Published:** 2022-01-14

**Authors:** Gabriel Broocks, Lukas Meyer, Rosalie McDonough, Matthias Bechstein, Uta Hanning, Jens Fiehler, Andre Kemmling

**Affiliations:** ^1^Department of Diagnostic and Interventional Neuroradiology, University Medical Center Hamburg-Eppendorf, Hamburg, Germany; ^2^Department of Neuroradiology, University Hospital Schleswig-Holstein, Lübeck, Germany; ^3^Department of Neuroradiology, University of Marburg, Marburg, Germany

**Keywords:** stroke, edema, thrombectomy, infarct, outcome

## Abstract

Randomized trials supporting the benefit of endovascular treatment in acute ischemic stroke patients with a large early infarction are not yet available. Few retrospective studies exist that suggest a potential positive treatment effect on functional outcome, as well as procedural safety. However, potential benefit or harm of MT in patients with low initial ASPECTS is still a subject of current debate, and in particular, how to select these patients for treatment. The purpose of this pilot study was to evaluate how early tissue water uptake in acute ischemic brain might determine lesion fate and functional outcome in low ASPECTS patients undergoing MT. We observed that the degree of early water uptake measured by quantitative NWU was significantly associated with functional outcome in low ASPECTS patients, yielding a higher diagnostic power compared to other parameters such as ASPECTS, age, or NIHSS. No conclusive evidence of a beneficial effect of successful reperfusion was observed in patients with low ASPECTS and high NWU, which highlights the potential of NWU as a tool to specify patient selection.

## Randomized Trials of Thrombectomy in Patients With Large Early Infarcts

Randomized clinical trial data supporting the benefit of endovascular treatment in acute ischemic stroke patients with a large early infarction are not yet available (1). Few retrospective studies exist that suggest a potential positive treatment effect on the functional outcome, as well as procedural safety (2–7). However, the potential benefit or harm of MT in patients with low inital ASPECTS is still a subject of current debate, and, in particular, how to select these patients for treatment. There is uncertainty of how to operationalize a threshold of extensive early infarct in CT to safely guide MT, which is reflected by the different inclusion criteria of the current low ASPECTS trials. TENSION and SELECT-2 include patients with ASPECTS 3–5, while LASTE (InExtremis) and TESLA include patients with 0–5 and 2–5, respectively (3–5 in LASTE for patients with > 80 years old) (8). Moreover, trials differ regarding the time window from the symptom onset to imaging, NIHSS cut-offs, or neuroimaging modality. The utilization of perfusion CT (CTP) as a selection tool has recently been critized with regard to overestimation of the true volume of irreversibly injured brain tissue, especially in the early time window, and in the context of the SELECT-2 trial (9–13). In particular, the exclusion of large core patients but ASPECTS >5 and/or early time window is controversial (9).

The rating of ASPECTS itself is based on binary scoring for presence of hypoattenuated brain tissue in 10 predefined territorial areas of the middle cerebral artery. This tissue hypoattenuation is directly related to early infarct with edematous water uptake, which can be quantified on CT (14). However, ASPECTS does not differentiate between different degrees of hypoattenuation in binary scored regions. Hence, the level of hypoattenuation (i.e., the level of water uptake indicated ischemic progression) may significantly differ across identical low ASPECTS ratings even after a similar time from the onset to imaging. It is thus conceivable that the potential benefit of MT in patients with low ASPECTS heavily depends on ischemic progression indicated by water uptake .

The purpose of this pilot study was to evaluate how early tissue water uptake in acute ischemic brain might determine lesion fate and the functional outcome in patients with low ASPECTS undergoing MT. We hypothesized that quantitative lesion water uptake measured in admission-CT differentiates patients with low ASPECTS with respect to the functional outcome after MT and predicts futile vessel recanalization.

## Pilot Study—Early Tissue Water Uptake as a Determinant of Response to MT

### Improving Low ASPECTS Stroke Thrombectomy (I-LAST)

I-LAST is an academic, independent, prospective, multicenter, observational registry study. This study aims to investigate the role of advanced imaging biomarkers in patients with large early infarct. The study is in accordance with the ethical guidelines of the local ethics committee and with the Declaration of Helsinki. The local ethics committee approved this study (WF-091/21) and waived informed consent. The study is registered within the ClinicalTrials.gov Protocol Registration and Results System (NCT04862507). The present pilot study used similar inclusion criteria (as stated below) and serves as an exploratory analysis to investigate the potential value of lesion water uptake as an imaging biomarker in the triage of patients with low ASPECTS.

### Patient Inclusion

For this exploratory pilot study, anonymized data from a tertiary-care stroke center were analyzed retrospectively.

All patients with acute ischemic stroke due to a large vessel occlusion in the anterior circulation admitted between March 2017 and March 2019 were consecutively screened for patients with (1) CT-angiography-confirmed occlusion of the M1 segment of the middle cerebral artery (MCA) or distal occlusion of the internal carotid artery (ICA); (2) admission multimodal CT protocol with non-enhanced CT (NECT), CT-Angiography (CTA), and Perfusion-CT (CTP) performed within 12 h from the symptom onset; (3) An initial ASPECTS of 5 or less in admission-NECT rated by a board-certified neuroradiologist. Decision for treatment was made by a board-certified neurointerventionalist in consensus with a board-certified attending stroke neurologist. Ischemic lesion net water uptake (NWU) was quantified as previously described (15, 16). A region of interest (ROI) was placed for density measurements according to the extent of ischemic hypoattenuation identified on NECT with hindsight knowledge of the core lesion in cerebral blood volume (CBV).

All finally analyzed patients were included in the regression analyses. Successful recanalization was defined as modified thrombolysis in cerebral infarctions (mTICI) score 2b-3. The patients who did not undergo MT were defined to have “no successful vessel recanalization.”

We compared the medians of the independent variables using Mann-Whitney U tests for patients based on the functional outcome at Day 90 after dichotomization in “good” (mRS 0–2) and “poor” outcomes (mRS 3–6). Receiver operating characteristic (ROC) curve analyses were performed to assess the area under the curve (AUC) of the independent variables [ASPECTS, NWU (continuous), age, and NIHSS] to compare its diagnostic ability to discriminate the functional outcome (dependent variable: mRS 0–2). Furthermore, a multivariable logistic regression analysis was performed using ASPECTS, NWU (continuous), age, time from the onset to imaging, NIHSS, and recanalization status as independent variables with stepwise variable selection. Moreover, “MT” was tested as an independent variable replacing “recanalization status.” The intention was to assess the independent association of NWU and the functional outcome, adjusted for covariates, especially ASPECTS, and recanalization status. We also tested the association of recanalization and the outcome separately for the patients with low and high NWU, distinguished using the NWU cut-off from ROC analysis. Finally, multivariable logistic regression analysis was performed with mRS 0–3 as a dependent variable, as the number of patients with functional independence (mRS 0–2) at Day 90 may be limited due to more distinctive baseline infarction.

A statistically significant difference was accepted at a *p*-value of < 0.05. Analyses were performed using MedCalc (version 11.5.1.0; Mariakerke, Belgium) and R (R Core Team. R: A Language and Environment for Statistical Computing. R Foundation for Statistical Computing. Vienna, Austria, 2017).

### Ethics Statement

Anonymized data were recorded and analyzed in accordance with ethical guidelines and after approval of the local ethics committee. Informed consent was waived.

## Results

**Figure 1:**
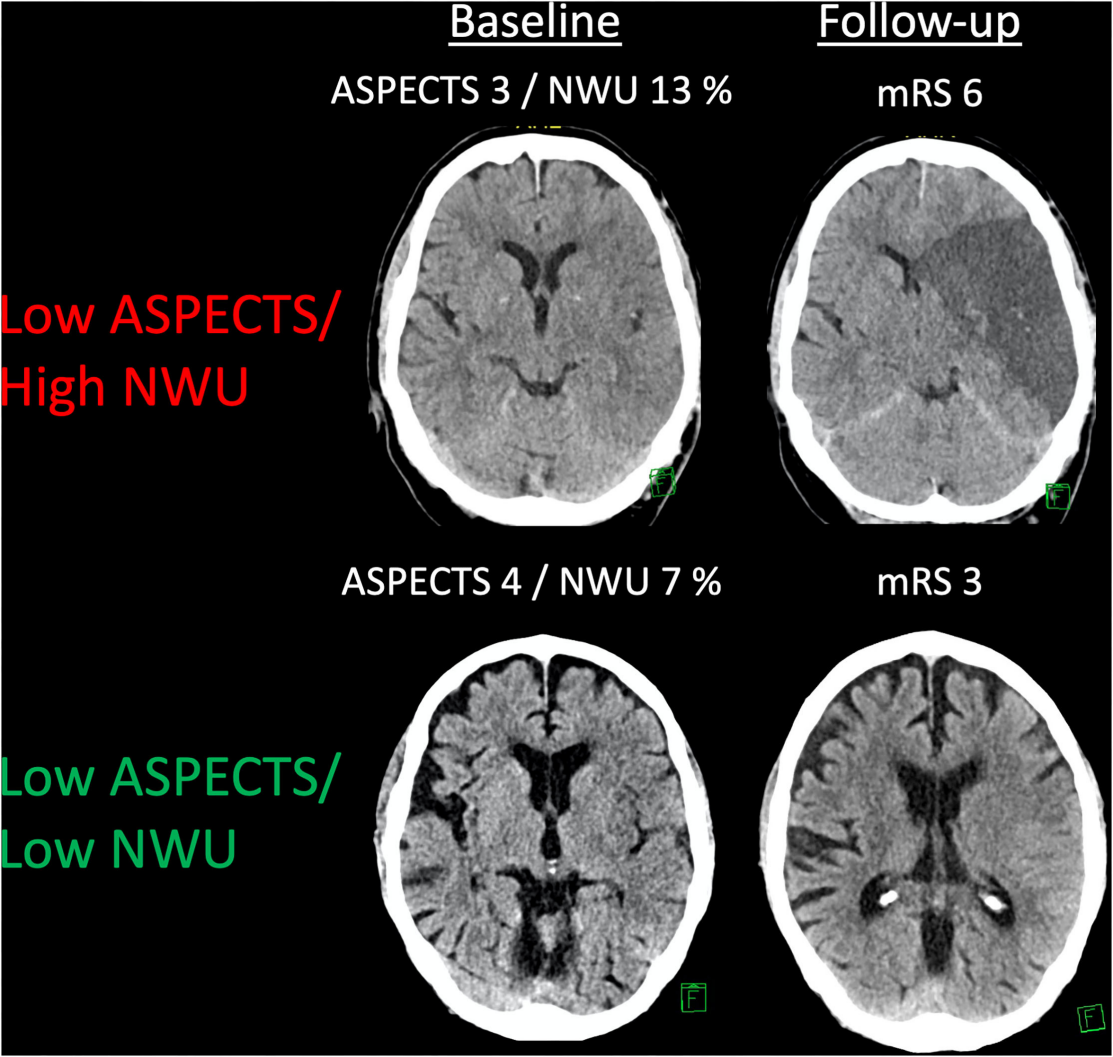
An example of patients with low ASPECTS and different kinds of net water uptake (NWU). Two examples showing patients with a low ASPECTS and low or high NWU, respectively. Follow-up imaging and a functional outcome are displayed.

About 155 patients were analyzed. The median ASPECTS was 4 (IQR: 3–5), and the mean NWU at admission was 10.8% (SD: 3.8%). The median age was 75 (IQR: 66–82). Seventy-eight patients (50.3%) underwent MT, of which 50 (64%) resulted in successful endovascular recanalization (mTICI ≥ 2b). The median mRS at Day 90 was 5 (IQR: 4–6) with 17 (11%) patients achieving functional independence. Figure 1 illustrates two examples of patients with low ASPECTS with high- vs. low-baseline NWU, respectively. Comparing the baseline NWU in patients with good clinical (mRS 0–2) vs. a poor outcome (mRS 3–6), the median NWU was significantly lower in patients with a good outcome (median 7.6, IQR: 4.6–8.7 vs. median 10.8, IQR: 8.9–13.9; *p* < 0.0001). Table 1 shows the patient characteristics.

**Table 1:** Patient characteristics.

**Variable**	***N* = 155**
Age (years)—median (IQR)	75.0 (66–82)
Time from onset to imaging in	3.8 (2.5–5)
hours—median (IQR)	
NIHSS—median (IQR)	18 (15–21)
ASPECTS—median (IQR)	4 (3–5)
NWU (%)–mean (SD)	10.8 (3.8)
IV rtPA, n (%)	88 (56.8)
Thrombectomy, n (%)	78 (50.3)
successful recanalization (mTICI ≥ 2b), n (%)	50 (64.0)
mRS90 0–2—n (%)	17 (11%)

**Figure 2:**
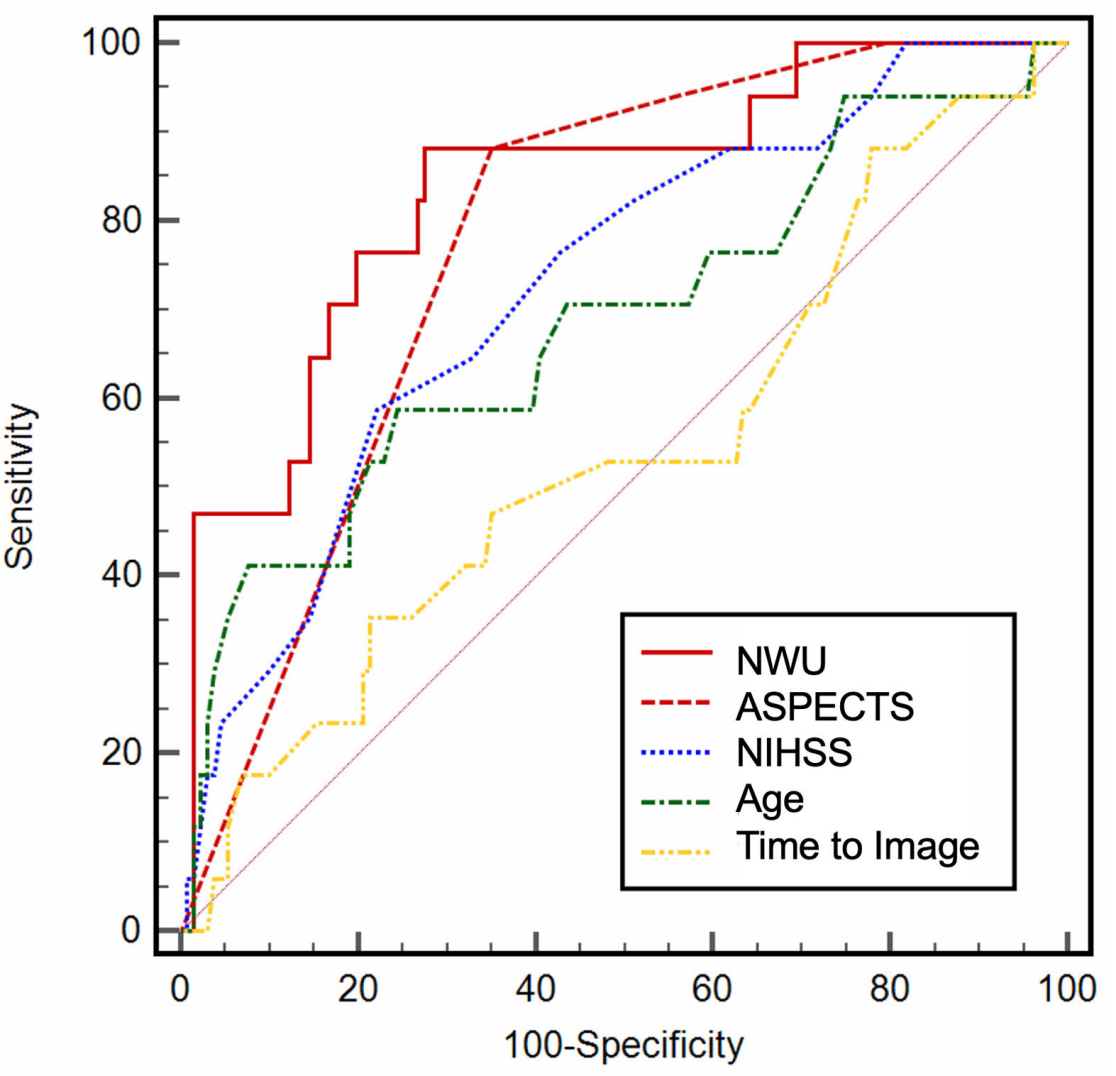
Receiver operating characteristic (ROC) curve analysis. ROC curve analysis for baseline variables, illustrating the diagnostic ability.

In ROC curve analysis, the highest diagnostic power was observed for NWU (AUC: 0.84; 95% CI: 0.77–0.89; Youden J: 0.59; cutoff, 9.1%; sensitivity, 88.2%; specificity, 70.6%), and ASPECTS (AUC: 0.78; 95% CI: 0.71–0.84; Youden J: 0.54; cut-off, 4; sensitivity, 88.2%; specificity, 65.4%). NIHSS on admission (AUC: 0.73) and age (AUC: 0.68) showed a moderate to low diagnostic ability to classify an outcome (Table 2; Figure 2). In multivariable logistic regression analysis, age (OR: 0.92; 95% CI: 0.85–0.99; *p* = 0.02), NWU (OR: 0.52; 95% CI: 0.37–0.74; *p* < 0.001), ASPECTS (OR: 17.89; 95% CI: 2.93–108.9; *p* = 0.001), and by trend status of recanalization (OR: 5.05; 95% CI: 0.97–26.5; *p* = 0.05) were significantly associated with a good functional outcome at Day 90 (Table 3). There was no significant interaction between NWU (continuous) and recanalization. The corresponding interaction term between NWU and recanalization status was not statistically significant (OR: 0.85; 95% CI: 0.55–1.29; *p* = 0.47). When replacing the independent variable “recanalization status” with “MT,” MT did not show a statistically significant association with an outcome (OR: 1.93; 95% CI: 0.28–13.26; *p* = 0.50).

**Table 2:** ROC curve analysis.

	**AUC**	**95%CI**
ASPECTS	0.78	0.70–0.84
%—Net Water Uptake	0.84	0.79–0.89
NIHSS on admission	0.73	0.65–0.80
Age	0.68	0.60–0.76
Time from onset—imaging	0.54	0.46–0.63

**Table 3:** Multivariable logistic regression analysis.

	**Modified rankin scale 0–2**			**Modified rankin scale 0–3**		
	**OR**	**95%CI**	***P* value**	**OR**	**95%CI**	***P* value**
NWU	0.52	0.37–0.74	<0.001	0.82	0.72–0.93	<0.01
ASPECTS	17.89	2.93–109	0.02	2.07	1.09–3.92	0.03
Age	0.92	0.85–0.99	0.02	0.95	0.92–0.99	0.01
Recanalization	5.05	0.97–26.5	0.05	n.s.	n.s.	n.s.

*n.s., not significant*.

In multivariable logistic regression analysis with mRS 0-3 as a dependent variable, NWU (continuous) was confirmed as an independent predictor of a good outcome (OR: 0.82; 95% CI: 0.72–0.93; *p* = 0.002), besides ASPECTS (OR: 2.07; 95% CI: 1.09–3.92; *p* = 0.03), and age (OR: 0.95; 95% CI: 0.92–0.99, *p* = 0.01) (Table 3).

In patients with NWU > 9.1% (cut-off from ROC curve analysis), successful recanalization did not show a statistically significant association with an improved outcome (OR: 1.17; 95% CI: 0.18–7.56, *p* = 0.87), while recanalization was significantly associated with a good outcome in patients with NWU < 9.1% (OR: 8.46; 95% CI: 1.7–41.1; *p* = 0.009).

## Discussion and Consequences for Clinical Trials

The aim of this pilot study was to investigate the impact of baseline NWU as a quantitative imaging biomarker in the assessment of patients with low ASPECTS. We hypothesized that the degree of hypoattenuation (i.e., water uptake) as a second lesion feature besides lesion extent modified the effect of MT on the functional outcome in patients with low ASPECTS. We observed that NWU was significantly associated with a functional outcome, yielding a higher diagnostic power compared to other parameters, such as ASPECTS, age, or NIHSS.

Yet, no randomized evidence supports MT for patients with low ASPECTS (17). Hence, diagnostic tools are needed to identify the patients with low ASPECTS that are expected to benefit from MT. The ASPECTS rating itself is based on binary subjective rating criteria (hypoattenuation yes/no). It, therefore, does not further quantify the degree of hypoattenuation, a major limitation of ASPECTS as a selection criterion for treatment. The stage of early infarct lesions in brain is defined by NWU, which, in turn, is directly related to lesion hypoattenuation and volume increase (i.e., extracellular edema). The decrease of attenuation of ischemic tissue is directly related to net influx of water, which has been demonstrated *in vitro* and *in vivo* (14, 18, 19). Therefore, an additional quantitative parameter that further stratifies lesion pathophysiology in patients with low ASPECTS could further differentiate the stages of early ischemic changes in acute stroke imaging to exclude those patients that do not benefit from MT (20).

In summary, we observed that neither MT nor successful recanalization after MT was associated with an improved outcome in patients with high NWU, adjusted for baseline ASPECTS. Hence, high NWU at admission may serve as a predictor of futile recanalization. In patients with low ASPECTS but low NWU, recanalization was significantly associated with functional independence. It is important to realize that the current trials investigating MT in patients with low ASPECTS do not consider lesion water uptake (i.e., the degree of hypoattenuation) as a factor for treatment selection, especially considering the extended time window from the symptom onset to imaging in these trials. Patient inclusion in TENSION requires a time from the onset to imaging of <11 h, while both TESLA and SELECT-2 include patients up to 24 h after the symptom onset. Hence, a patient with an ASPECTS of 2, high degree of lesion water uptake, and presenting after up to 24 h after the onset may be randomized in the TESLA trial and undergo MT. According to our data, recanalization of patients with low ASPECTS with high water uptake is not associated with a clinical benefit, and futile MT may occur with high probability. Moreover, ASPECTS remained an independent predictor of an outcome despite preselection of patients (i.e., ASPECTS 0–5 only) with a high OR, which emphasized differences among the lower ASPECTS scale, particularly encouraging treatment of ASPECTS 4–5.

Alternative concepts to patients with triage low ASPECTS are currently discussed, for instance, the utilization of CTP (5). This approach, however, is often criticized, in particular with regard to overestimation of the volume of irreversibly injured tissue, potentially causing the exclusion of patients who might benefit from MT (5, 11, 21). Second, CTP-derived core volumes did not modify the effect of MT on a clinical outcome in a HERMES meta-analysis (22). In contrast, NWU could serve as a quantitative imaging biomarker that specifically represents ischemic edema as a hallmark of irreversible infarction with very low likelihood of reversibility (5, 14, 23).

An approach for a clinical feasible implementation of NWU in the assessment of patients with extensive baseline infarct lesion could be realized by an adjusted ASPECTS. The degree of hypoattenuation per ASPECTS region could be quantified using simple measurements of relative density changes in the ischemic brain tissue compared to a contralateral region of interest and then be used to adjust the ASPECT score. Further studies are needed to validate the impact of NWU in patients with low ASPECTS and to test the applicability and diagnostic ability of an edema-corrected ASPECTS. Limitations of our study include the relatively small number of patients due to strict inclusion criteria. Furthermore, the retrospective design of this study does not allow for valid conclusions regarding treatment effects. Moreover, the matching of patients who did not undergo MT as “not successfully recanalized” might lead to an underestimation of the effect of MT due to the potential phenomenon of spontaneous reperfusion. We did not observe a significant interaction between baseline NWU and recanalization, but this might be biased by the small number of patients. Yet, the quantification of NWU in admission CT requires the utilization of CTP, which is a limitation of this method. Future studies should validate alternative concepts of NWU quantification, for instance, using automated voxel-wise analysis of NECT hypoattenuation per ASPECTS region, or using multiphase CTA maps to define the ROI for NWU quantification in NECT.

## Conclusion

In stroke patients with LVO and initial ASPECTS of ≤ 5, low NWU of early infarct may constitute a beneficial constellation for vessel recanalization. No conclusive evidence of a beneficial effect of successful reperfusion was observed in patients with low ASPECTS and high NWU, which highlights the potential of NWU as a tool to specify patient selection.

## Data Availability Statement

The datasets presented in this article are not readily available due to ethical restrictions that prevent the sharing of data. Requests to access the datasets should be directed to the corresponding author.

## Ethics Statement

The studies involving human participants were reviewed and approved by Ethikkommission der Ärztekammer Hamburg. The Ethics Committee waived the requirement of written informed consent for participation.

## I-Last Study Collaborators

Gabriel Broocks, Department of Neuroradiology, University Medical Center Hamburg Eppendorf, Germany; Tobias D. Faizy, Department of Neuroradiology, Stanford University, Stanford, United States; Andre Kemmling, Department of Neuroradiology, University Hospital, Marburg, Germany; Sönke Langner, Department of Neuroradiology, University Medical Center, Rostock, Germany; Andre Kemmling, Department of Neuroradiology, University Hospital Schleswig-Holstein, Luebeck, Germany; Jawed Nawabi, Department of Radiology – Charite University Medicine, Berlin, Germany; Panagiotis Papanagiotou, Department of Neuroradiology Klinikum Bremen-Mitte GmbH, Bremen, Germany; Peter Sporns, Department of Neuroradiology, Universitätsspital Basel, Basel, Switzerland.

## Author Contributions

GB, LM, JF, and AK have contributed in conception and design of the study. GB, RM, MB, and AK have contributed in acquisition and analysis of data. GB, UH, JF, and AK have contributed in drafting a significant portion of the manuscript. All authors contributed to the article and approved the submitted version.

## Conflict of Interest

JF receives research support from EU, BMBF, BMWi, DFG, Acandis, Medtronic, Microvention, and Stryker. JF is a consultant for Acandis, Codman, Cerenovus, Medtronic, Microvention, Penumbra, Phenox, and Stryker, holds Stock of Tegus Medical, and has executive functions at Eppdata (all unrelated). AK has research collaboration agreement with Siemens Healthcare (unrelated).

The remaining authors declare that the research was conducted in the absence of any commercial or financial relationships that could be construed as a potential conflict of interest.

## Publisher's Note

All claims expressed in this article are solely those of the authors and do not necessarily represent those of their affiliated organizations, or those of the publisher, the editors and the reviewers. Any product that may be evaluated in this article, or claim that may be made by its manufacturer, is not guaranteed or endorsed by the publisher.
